# Real-Time Single Frequency Precise Point Positioning Using SBAS Corrections

**DOI:** 10.3390/s16081261

**Published:** 2016-08-10

**Authors:** Liang Li, Chun Jia, Lin Zhao, Jianhua Cheng, Jianxu Liu, Jicheng Ding

**Affiliations:** 1 College of Automation, Harbin Engineering University, Harbin 150001, China; zhaolin@hrbeu.edu.cn (L.Z.); chengjianhua@hrbeu.edu.cn (J.C.); liujianxu@hrbeu.edu.cn (J.L.); dingjicheng@hrbeu.edu.cn (J.D.); 2 Acdemy of Opto-electroncis, Chinse Academy of Sciences, Beijing 100094, China

**Keywords:** precise point positioning, SBAS, real-time, single frequency, convergence

## Abstract

Real-time single frequency precise point positioning (PPP) is a promising technique for high-precision navigation with sub-meter or even centimeter-level accuracy because of its convenience and low cost. The navigation performance of single frequency PPP heavily depends on the real-time availability and quality of correction products for satellite orbits and satellite clocks. Satellite-based augmentation system (SBAS) provides the correction products in real-time, but they are intended to be used for wide area differential positioning at 1 meter level precision. By imposing the constraints for ionosphere error, we have developed a real-time single frequency PPP method by sufficiently utilizing SBAS correction products. The proposed PPP method are tested with static and kinematic data, respectively. The static experimental results show that the position accuracy of the proposed PPP method can reach decimeter level, and achieve an improvement of at least 30% when compared with the traditional SBAS method. The positioning convergence of the proposed PPP method can be achieved in 636 epochs at most in static mode. In the kinematic experiment, the position accuracy of the proposed PPP method can be improved by at least 20 cm relative to the SBAS method. Furthermore, it has revealed that the proposed PPP method can achieve decimeter level convergence within 500 s in the kinematic mode.

## 1. Introduction

The precise point positioning (PPP) technology relies on external correction products for satellite orbits and clock errors [1,2,3,4], as well as the ionosphere error which is optional depending on the availability of dual frequency observations. Real-time PPP has evolved as a powerful technique to achieve globally homogenous position accuracy [5,6], which can be implemented based on either dual frequency or single frequency mode [7]. For most real-time navigation or geo-referencing applications, only single frequency GNSS receivers are used due to their low-cost [8,9], therefore, the high precision real-time single frequency PPP has attracted great attention [10]. Presently, single frequency PPP is able to provide centimeter level accuracy in static mode and decimeter level in kinematic mode [2].

The convergence time is an important factor affecting the real-time performance of PPP. The PPP solution convergence time ranges from several minutes to a few hours depending on both the expected position accuracy and the mode of receiver operation. The convergence time can significantly be reduced by fixing the carrier phase ambiguities [11,12,13]. This, however, requires additional correction products on signals delays, for instance the corrections of uncalibrated phase delays (UPD) [14], which are currently difficult to obtain real-time.

The navigation performance of single frequency PPP depends on the quality of correction products such as the real-time availability, correction and precision. One of the most commonly used correction products provided for satellite orbit and clock errors is the International GNSS service (IGS) from which correction products are available with different latencies, ranging from 3 h for ultra-rapid, to 17 h for rapid, and 13 days for the final products [3,10]. With the rapid development of PPP, a broad need of real-time PPP applications are arising, such as precision agriculture, precise maritime navigation, and hydrography. Recently, the predicted ultra-rapid products satellite clock with a latency of a few seconds has been made available through the Internet by IGS [3], or the real-time products is broadcasted by INMARSAT satellites provided by some commercial companies such as Fugro and Trimble. However, the real-time performance of the former one cannot be guaranteed because of the unpredicted internet congestion, and the latter one requires high cost for providing continuous and reliable products.

In order to implement the real-time single frequency PPP, the satellite orbit and clock correction, as well as the ionospheric error correction have to be obtained in real-time. One possible way to acquire real-time correction products is the usage of wide-area real-time kinematic (WARTK) results [15,16,17], but this method is limited to land applications because it is difficult for maritime or aerospace receivers to obtain the correction products from WARTK network.

Another means by which corrections are disseminated by geostationary (GEO) satellites are the satellite-based augmentation systems (SBAS). The typical implementations include wide area augmentation system (WAAS), the European geostationary navigation overlay service (EGNOS), the multi-functional satellite augmentation system (MSAS), and satellite differential correction and monitoring system (SDCM). Since SBAS broadcasts real-time correction products of ionosphere error, satellite orbits and clocks, which are the main error sources to be corrected for PPP, SBAS can provide positioning services of 1–2 m accuracy level with high integrity level, which is essential for safety-critical applications such as civil aviation [18]. If we use the real-time SBAS products for PPP, real-time wide area PPP can be realized. Although the accuracy of satellites’ orbit and clock correction products provided by SBAS is relatively lower than the post-processing precise correction products, more importantly, the SBAS products can be obtained in real time. Efforts utilizing SBAS corrections for PPP have been made by Rho and Langley [16], as well as Heßelbarth and Wanninger [1]. They both performed carrier-phase-based PPP by using dual frequency observations. The single frequency PPP performance using SBAS corrections has however not been analyzed yet. Furthermore, the real-world kinematic positioning performance has also not been evaluated yet. Therefore, the purpose of this contribution was to develop and test a real-time single frequency PPP, by using the real-world static and kinematic data simultaneously, which is the extension of previous research. Since we focus on the feasibility and performance analysis of single frequency real-time PPP, we are not going to discuss in detail the effect of various SBAS systems corrections on the proposed method in this paper.

Compared with the dual frequency PPP using SBAS corrections, there are many issues for single frequency PPP to solve: firstly, without the ionosphere-free observation from dual frequency combination, the ionosphere error has to be dealt with in the case of single frequency. Secondly, because of the relatively low precision of SBAS correction products, the observation biases have to be modeled or estimated more accurately to achieve high precision positioning results. This may increase the convergence time and induce the position estimation model to be rank-deficient. On the other hand, there are also at least two advantages in the case of single frequency PPP. The prime advantage is that the observation noise level (approximately 1/3 times) lower than the one of ionosphere-free combination. The other advantage is the promising prospect of single-frequency PPP because of its low-cost. These challenges and promising future stimulate this research.

The focus of this paper is a real-time single frequency PPP approach with real-time SBAS corrections. By eliminating and/or modelling the satellite related error and the atmospheric error, we will present a detailed PPP coordinate estimation algorithm with the accurate characterization of stochastic model. The performance of the proposed single frequency PPP approach will be tested by using the static data from 12 permanent stations, and the kinematic data from the suburban environment, respectively. Finally, the conclusions and remarks will be summarized.

## 2. Observation Error Correction

Since we focus on the real-time single frequency PPP using SBAS corrections, the single frequency observation model and the features of SBAS correction products for different error sources are analyzed in this section.

### 2.1. Observation Functional Model

In the case of single frequency GPS receiver, the code and carrier-phase observation are normally available. They can be expressed as:

(1) $$p=\rho +c(dt_{r}-dt^{s})+I+T+d_{orb}+b_{p}+\xi +\varepsilon_{p}$$

(2) $$\varphi =\rho +c(dt_{r}-dt^{s})-I+T+d_{orb}+n+b_{\varphi }+\xi +\omega_{\varphi }+\varepsilon_{\varphi }$$

where *p* and *ϕ* are the observed code and carrier phase, *ρ* is the geometric range between receiver and satellite, *dt_r_* and *dt^s^* are the receiver and satellite clock errors respectively, *I* is ionosphere delay, *T* is tropospheric delay, *d_orb_* is satellite position geometric error, *n* is integer ambiguity in meters, *c* is the speed of light, *b_p_* the differential code bias (DCB), *ω_ϕ_* the phase wind-up effect, *b_ϕ_* the hardware phase delay, *ξ* represents the measurement biases relating to the site displacement effects (solid Earth tide, ocean loading, pole tide), relativity and Sagnac effect, satellite and receiver-antenna phase offsets and variations, *ε_p_* and *ε_ϕ_* contain the unmodeled quantities such as observation noise and multipath, specific to observation. A detailed description of all these effects is given by Kouba [3]. The real-time single frequency PPP uses several public observation error correction products and models to account for some error sources, such as Saastamoinen model for troposphere [19].

The more observation error sources are corrected, the higher position accuracy and the faster convergence can be obtained. One set of SBAS message types include various correction components for satellite-generated errors, including separate corrections for the one satellite clock error state and the three axes of satellite orbit error, for all satellites covered by SBAS at a given time. Meanwhile, a completely separate set of SBAS message types contain the correction coefficients of ionospheric error [20,21,22]. However, the precision of real-time correction products from SBAS are relatively lower than that of IGS. The differences between the correction products of SBAS and IGS require a modification of the observation error correction strategy for the real-time single frequency PPP using SBAS corrections, which will be analyzed next.

### 2.2. Satellite Orbit and Clock Error Correction

Unlike precise ephemeris from IGS, SBAS broadcasts the corrections to the satellite orbit and clock error with respect to the broadcast ephemeris. The correction products of satellite orbit and satellite clock can be divided into the long-term and fast corrections. The long-term corrections contain information on the slowly varying satellite orbit and clock errors, whereas the fast corrections provide information on the fast varying clock errors. The calculate formulas of SBAS satellite orbit and clock error correction can be written as:

(3) $$r_{SBAS}=r+\dot{r}\Delta t$$

(4) $$\delta \Delta t_{sv}=\delta a_{f0}+\delta a_{f1}\Delta t+PRC/c$$

where **r***_SBAS_* is the satellite coordinate correction vector, Δ*t* is the satellite clock correction error estimate, **r** is the satellite correction with respect to SBAS ephemeris time, $\dot{r}$ is the rate-of-change vector of satellite position, *δa_f_*_0_ and *δa_f_*_1_ is the satellite clock offset error correction and the clock-drift error correction coefficients, *PRC* is fast clock varying corrections [20]. By using the satellite orbit and satellite clock corrections, the errors of satellite orbit and satellite clock with respect to the broadcasted emphatics can be greatly suppressed.

The resolution of long-term satellite orbit corrections and also of the fast corrections is 12.5 cm, which is lower than IGS products and limits the achievable accuracy of the PPP. The relatively low precision of correction products inhibits the ambiguity resolution for PPP using SBAS. It therefore can be anticipated that PPP using IGS products have better positioning performance than that using SBAS corrections. When using SBAS corrections for PPP, there are some differences in the definition of various satellite orbit and clock products that must be taken into account. One difference is the correction reference point at the satellite. GPS broadcast ephemeris refer to the center of the satellite antennas [23], while IGS products are based on the center of mass of the satellite [3]. Since the observations are made to the apparent satellite antenna phase center of the ionosphere-free combinations of dual frequency observations, corrections must be introduced, which have to be identical to those used by the IGS. There will be a position bias of 4 cm without applying these corrections contained in IGS files, e.g., igs08.atx. The research of Heßelbarth and Wanninger [1] has found that the WAAS satellites phase center also has to be corrected, but the details for EGNOS and MSAS satellites phase center are still not clear. We therefore disregard the difference in this paper. There are also other differences between different SBASs such as the geodetic references frame. In this research, we use the WGS84 as the position coordinate reference system.

### 2.3. Atmospheric Error Correction

The atmospheric error for PPP includes two parts, i.e., the ionospheric and the tropospheric error. Both IGS and SBAS have provided specific correction products or estimating models for ionospheric and tropospheric errors.

IGS correction products for ionosphere known as global ionospheric model (GIM) can be used for precisely compensating ionosphere errors. However, the use of GIM models is also limited by the issue of real-time availability because of its significant latency. In contrast, SBAS broadcasts real-time ionosphere delay correction parameters. The estimated delay corrections for a given satellite due to the ionosphere at the receiver can be computed using the four corners of the ionospheric grid points (IGP) geographic box in which the receiver is located to determine a slant delay correction. The IGP locations are denser at lower latitudes because of the fact that the distance represented by a degree of longitude becomes smaller at higher latitudes. Therefore, it can be anticipated that the receivers at low latitudes will obtain higher position accuracy than the ones at high latitude.

The ionosphere remains a major bias for single frequency PPP that may be eliminated with a group and phase ionospheric calibration (GRAPHIC) linear combination [24]. However, because the noise level of GRAPHIC combination is dominated by the code observation noise, the position coordinate and ambiguity parameters therefore cannot be determined using a single epoch of observations due to the rank-deficient issue [25,26]. An estimation process using cumulative observations has to be applied and a long time period of 2–4 h are also required for the float ambiguity parameters to converge [27]. Because this research focuses on real-time applications, thus, the GRAPHIC model will not be used.

Since the ionosphere delay is the dominant error, a more precise characterization model is positive to suppress the ionospheric error. It has been proven that the ionospheric error can be modeled by tilting the zenith of ionosphere to estimate the ionospheric gradients along with zenith delay [25]:

(5) $$d_{ion}=I_{z}M_{F}+M_{F}\cot e\cos\alpha g_{n}+M_{F}\cot e\sin\alpha g_{e}$$

where *d*_ion_ is the ionospheric correction obtained from SBAS. *e* is the elevation angle and α is the azimuth angle. *I_z_* is the zenith delay, *g_n_* and *g_e_* are the horizontal gradients in the north and east directions, respectively. *M_F_* is the mapping function and can be computed as *M_F_* = [1−cos^2^e/(1+*h*/*R*)^2^]^−1/2^, in which *R* is the mean radius of the Earth and *h* is the average height of the ionosphere layer [28]. The uncertainty of ionospheric error is modeled as a function of variance of ionospheric delay at the IGPs [20].

Tropospheric error can be separated into a dominate hydrostatic part and a much smaller wet part, in which the hydrostatic part can be molded and considered be known, while the wet part has to be estimated. Therefore, one candidate method for troposphere error estimation is to introduce the zenith tropospheric delay combined with mapping function [3]. SBAS has also recommended the troposphere model to compensate the hydrostatic and wet part simultaneously by using off-line meteorological parameters [20,29]. The residual tropospheric error is weighted as a function of elevation. In order to precisely characterize the troposphere error, we use the SBAS recommended model and estimate the zenith delay of wet troposphere residual.

Besides the satellites’ orbit and clock errors, and the atmospheric error, there are many other errors to be corrected/modelled in order to further improve the position accuracy of PPP, as seen from the observation model of (1) and (2). However, there are some errors, for instance the site displacement effects, that need external correction parameters. These corrections are presently available with different latencies. Particularly, these corrections do not included in the standardization of SBAS. Therefore, the satellite orbit and clock error, and the atmospheric error are chosen to be corrected for single frequency PPP. The other errors will be considered as unmodelled observation error.

## 3. Positioning Algorithm

With the corrected single frequency code and carrier-phase observations, the user coordinates can be precisely resolved. Let us assume that there are *s* satellites available and no cycle slips in phase observations, the processing strategy for the observation error sources for PPP is listed in Table 1. The Gaussian-Markov model for position estimation can be written as:

(6) $$E\left[\begin{array}{c} v_{p}\\ v_{\varphi }\\ d_{ion} \end{array}\right]=\left[\begin{array}{cccc} G & M_{T} & M_{I} & 0\\ G & M_{T} & -M_{I} & I\\ 0 & 0 & M_{I} & 0 \end{array}\right]\left[\begin{array}{c} x\\ \beta \\ \iota \\ n \end{array}\right]\to E\left(L\right)=H\Delta$$

where ***v****_p_*, ***v****_ϕ_* are the *s* × 1 code and phase observation residual, ***d****_ion_* is the *s* × 1 ionosphere correction from SBAS. The ***x***, ***β***, and ***n*** are the corrections to the a priori parameters: the corrections to the position coordinates ***x*** = [*δe δn δu*]^T^ with the *s* × 3 observation matrix ***G***, the corrections to the zenith delay of wet troposphere residual and receiver clock bias ***β*** = [*δT_z_ δt_r_*]^T^ with the *s* × 2 design matrix ***M****_T_*, ***n*** is the *s* × 1 corrections to the ambiguities. ***ι*** is the ionosphere gradients parameters [*I_z_* *g_e_* *g_n_*]^T^ with the *s* × 3 design matrix ***M****_I_* from Equation (5), ***I*** is the identity matrix with the rank *s*, ***0*** is the *s* × *s* matrix of zeros. It can be found that the instantaneous position estimation can be implemented when the available satellites are more than 4. The Gaussian-Markov model from Equation (6) can be rank-deficient without the ionosphere constraint from Equation (5).

The atmospheric corrections are usually treated as deterministic quantities. This is however unrealistic since the estimated atmospheric corrections obtained from SBAS are random and furthermore the interpolated corrections diverge from the realistic corrections. This will cause the cross-correlation between the code and phase observations. We assume the observation regarding to satellites are independent with each other, the stochastic model of the proposed model (6) can be characterized as:

(7) $$R=D\left\{\left[\begin{array}{c} v_{p}\\ v_{\varphi }\\ d_{ion} \end{array}\right]\right\}=\left[\begin{array}{ccc} \sigma_{UDRE}^{2}+\sigma_{trop}^{2}+\sigma_{p}^{2} & \sigma_{UDRE}^{2}+\sigma_{trop}^{2} & 0\\ \sigma_{UDRE}^{2}+\sigma_{trop}^{2} & \sigma_{UDRE}^{2}+\sigma_{trop}^{2}+\sigma_{\varphi }^{2} & 0\\ 0 & 0 & \sigma_{UIRE}^{2} \end{array}\right]⊗I$$

where D{·} denotes the dispersion operator, ⊗ is the Kronecker product operator, *σ_trop_* is the STD of residual troposphere error, *σ_UDRE_* is the STD of orbit and clock correction residual, *σ_p_* and *σ_ϕ_* are the elevation-dependent STD of code and observation noise including the effect of other uncharacterized observation bias, *σ*_UIRE_ is the STD of ionosphere correction error. Note that *σ_trop_*, *σ_UDRE_*, and *σ_UIRE_* follow the recommended calculating formulas of SBAS [20].

With the proposed mathematical model (6) and stochastic model (7), we can use the sequential least-squares as introduced in [3] to calculate the receiver position coordinate:

(8) $$\hat{\Delta }={\left(Q_{\Delta }+H^{T}R^{-1}H\right)}^{-1}H^{T}R^{-1}L$$

where $Q_{\Delta }$ is the variance-covariance matrix for the unknown parameters ***Δ***. It is noted that the a priori parameters setup for the ambiguity has to be reinitialized if the cycle slip is detected. The alert for cycle slip can be raised if the epoch differenced of test statistic *t = p*−*ϕ* is beyond the protection of detection threshold *T* = 4.2*σ_p_*.

## 4. Experiments and Discussion

In order to sufficiently test the real-time PPP performance using the SBAS correction products, we carried out static and kinematic experiments, respectively. The proposed PPP method (denoted as SBAS-PPP) is compared with the SBAS-based positioning using single frequency code observation (denoted as SBAS), and the single frequency PPP using IGS final products (denoted as IGS-PPP) in order to reveal the extreme performance of the proposed SBAS-PPP method. The float solutions of IGS-PPP, i.e., without fixing carrier phase ambiguities, are chosen because the UPD products are difficult to achieve in real-time. The ionospheric corrections for SBAS-PPP result from SBAS, while the GIM products from IGS is utilized for IGS-PPP. The position performance is evaluated by the metrics of position accuracy and convergence time. It is noted that the 95th percentile of position errors is selected to indicate the position accuracy, and the convergence time is defined as the amount of GPS data, i.e., time span of GPS data, required for coordinate to be estimated with the horizontal and vertical position errors less than the predefined thresholds. The elevation-dependent weighting is applied, and the observation standard deviation (STD) at elevation θ is σ_o_ = σ_z_(1 + 1/sinθ) with σ_z_ being the STD in zenith, which takes 5 mm and 50 cm for carrier phase and code, respectively. The cut-off elevation angle is set as 10 degrees. Note that the datasets were processed after the fact, i.e., post-processed, but ‘‘as if’’ in real-time (re-played), strictly with public SBAS correction products available at the time of observation.

### 4.1. Static Positioning Solutions

**Figure 1:**
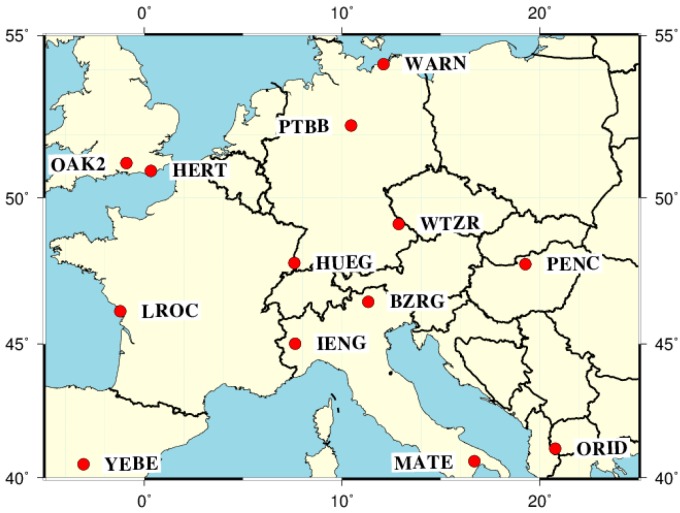
European stations used in the static experiment.

Since the EGNOS data are publically available at www.egnos-pro.esa.int/ems/index.html, we therefore selected 12 IGS permanent stations from Europe, which are shown in Figure 1. Their observation data are made publicly available by IGS, and their IGb08 coordinates can be precisely known. The coordinate difference between IGb08 and WGS84 are disregarded at the experiments. For each of these stations, single frequency code and carrier-phase observation data sets of 10 consecutive days were evaluated, the sampling period of the static observation data is 30 s, which yields 28,800 epochs for each station. The predefined positioning thresholds for horizontal and vertical convergence are set as 30 cm and 50 cm, respectively.

**Figure 2:**
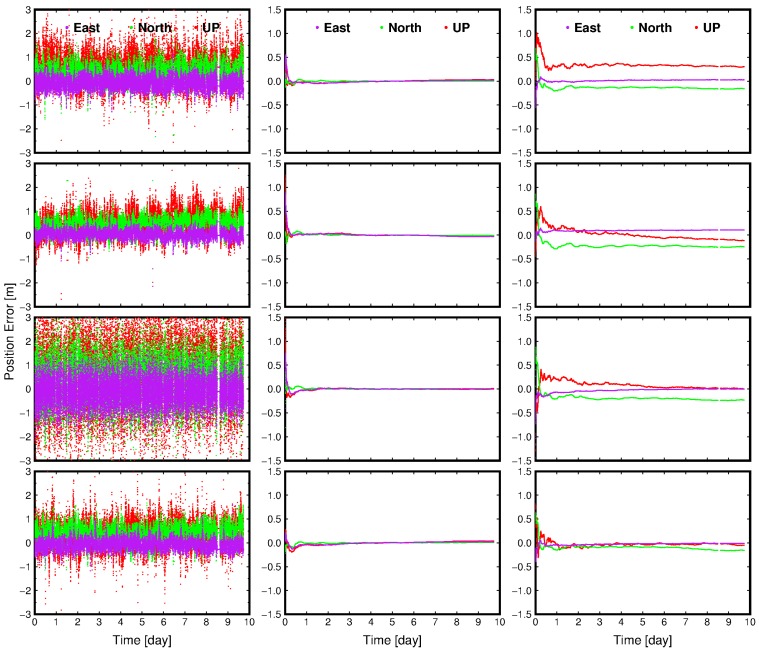
Dispersion of position coordinates for four permanent stations using different positioning algorithms. The column panels from top to bottom represent the position results from by using the positioning algorithms of SBAS, IGS-PPP, SBAS+PPP, respectively. The row panels from left to right represent the position result for four stations, i.e., PENC, PTBB, HERT, BZRG, respectively.

Figure 2 presents the positioning results of four example permanent stations as a function of time. It can be seen that the position error of SBAS ranges from 1 m to more than 3 m, and are quite diverse for the four stations. Furthermore, it can be found that the SBAS-PPP can achieve the convergence of position accuracy at decimeter level, which is remarkable higher than the one of SBAS, regardless of the locations of stations. In contrast, the position accuracy IGS-PPP can achieve is centimeter level, which is higher than SBAS-PPP. When the position error of SBAS-PPP is converged, it can be found that the position accuracy for SBAS-PPP is only a few centimeters lower than IGS-PPP, which indicates the precision of SBAS correction products is lower than IGS, and thus a longer convergence time of SBAS-PPP is required to achieve a comparable position accuracy of IGS-PPP.

The numerical statistics of position error for 12 permanent stations are shown in Table 2. Since all the permanent stations are located at mid-latitude sites, it can be found that the SBAS provides the position accuracy with the 1–3 m level. In contrast, the position accuracy of SBAS-PPP is remarkable higher than that of the SBAS. Specifically, the highest horizontal (12.1 cm) and vertical position accuracy (12 cm) of SBAS-PPP can be obtained at stations IENG and HUEG, while the station OAK2 has lowest horizontal position accuracy of 30.5 cm, station MATE with the lowest vertical position accuracy of 55 cm. In contrast, the position accuracy of IGS-PPP is generally higher than SBAS-PPP. The highest horizontal (3.3 cm) and vertical position accuracy (1 cm) of IGS-PPP can be observed at stations HUEG and YEBE, respectively, and the lowest horizontal (10.6 cm) and vertical position accuracy (8 cm) can be obtained at stations MATE and PENC. Among these three methods, the position accuracy of SBAS is the lowest one because the low precision code observations are used, which illustrates the necessity of utilizing carrier phase observations for precise positioning. Although the position accuracy of IGS-PPP is the highest among these three methods, the SBAS-PPP can achieve the position accuracy of several decimeters, which illustrates the ability of real-time single frequency PPP using SBAS corrections in the static mode.

**Figure 3:**
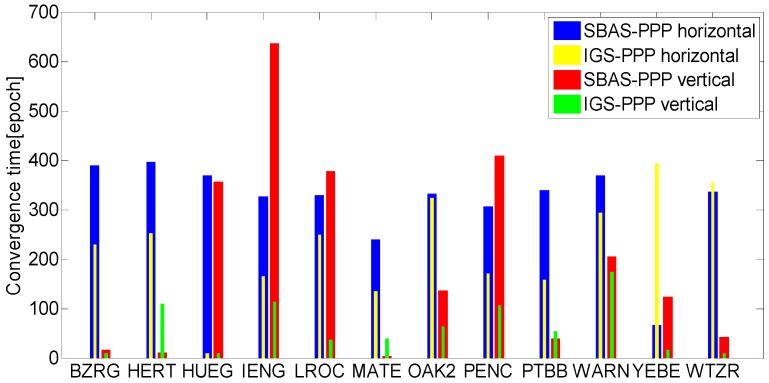
Convergence time for 12 stations.

In order to investigate real-time performance of SBAS-PPP, the position solution convergence time of SBAS-PPP and IGS-PPP are compared and presented in Figure 3. The convergence time of SBAS is not presented because it is difficult for SBAS to satisfy the predefined threshold for determining convergence. The convergence analysis are based on 6 h of static data, which yields 40 datasets to analyze the convergence performance. According to the figure, the average convergence time for SBAS-PPP needs hundreds of epochs for both horizontal and vertical positioning, in which the shortest convergence time for SBAS-PPP is obtained at stations YEBE (horizontal) and HERT (vertical), and the longest convergence time is obtained for stations HERT(horizontal) and IENG (vertical). The horizontal convergence time of IGS-PPP vary from tens to hundreds of epochs for different stations. It is noted that the horizontal convergence time of IGS-PPP are shorter than SBAS-PPP, except for stations YEBE and WTZR. The shorter convergence time performance of IGS-PPP can be explained by twofold: One is the higher precision of correction products. The other is the observation biases have been more accurately corrected or modelled by the proposed estimating and weighting model.

### 4.2. Kinematic Positioning Solutions

**Figure 4:**
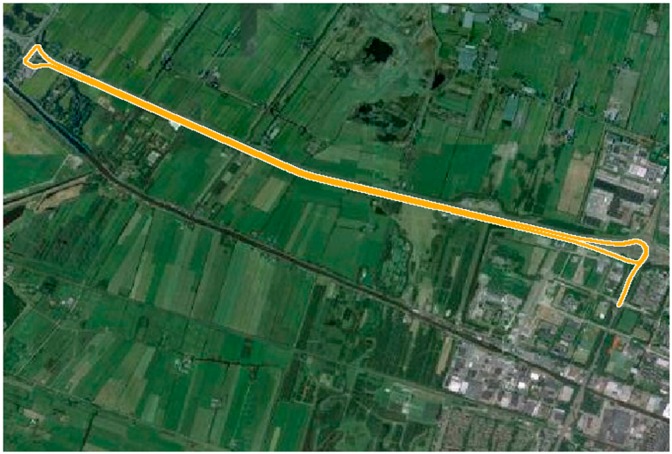
Trajectory of rover receiver.

The kinematic experiment was carried out in suburs of Delft (The Netherlands). The kinematic PPP experiment was processed by combining with EGNOS. As induced from the static experiment, the over-long convergence time of SBAS-PPP is negative for the real-time navigational performance. The sampling period during the kinematic experiment was set as 0.1 s. The trajectory of the rover receiver is shown in Figure 4. In order to evaluate the kinematic experimental result, the RTK positioning result in the ambiguities-fixed mode, which came from a commercial post-processing software NovAtel GrafNav, were used as the positioning reference with an accuracy of 5 cm (1σ) as claimed by GrafNav. A total number of 16,000 epochs of data were collected.

**Figure 5:**
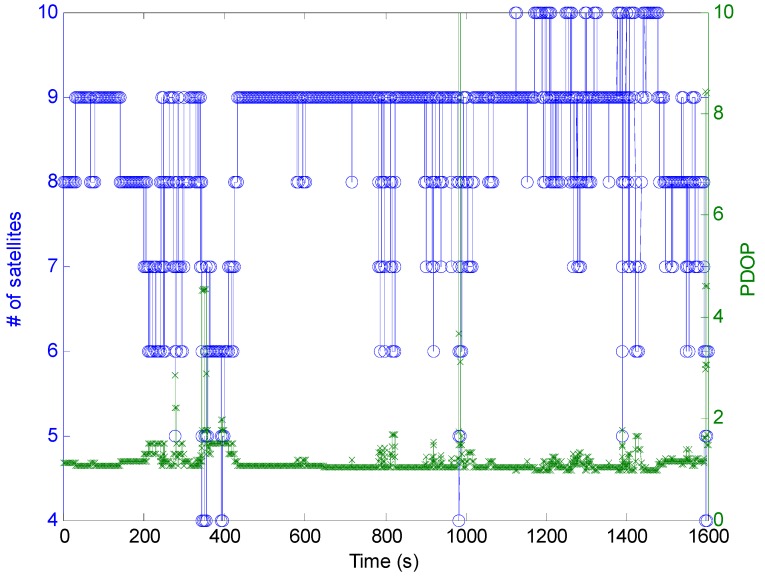
Available satellites and PDOP during the kinematic test.

**Figure 6:**
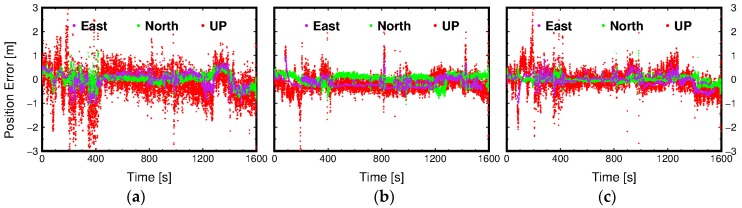
Position errors using different methods. The panels from left to right are the position errors of (**a**) SBAS; (**b**) IGS-PPP; and (**c**) SBAS-PPP, respectively.

Figure 5 shows the number of available satellites and PDOP during the kinematic experiment. Figure 6 presents the position errors in each positioning component using different positioning methods. It can be found that position errors of all three positioning methods increase when the PDOP is larger. Through combining Figure 5 and Figure 6, it can be observed that the position errors of three methods are dramatically increasing especially when the number of available satellites are less than five. Under the challenging kinematic experimental environment, there are eleven positioning re-initialization operations because the available satellites were less than four after the quality control of observations.

The position accuracy statistics for SBAS, IGS-PPP, and SBAS-PPP are shown in Table 3. From Figure 6, we can see that there are multiple peaks of the position errors for three methods because there are multiple positioning re-initialization operations as stated above. Furthermore, it can be found that the SBAS method has the lowest position accuracy, and the position accuracy of IGS-PPP is the highest among these three method, which can be demonstrated by numerical position error comparison in Table 3.

As shown in Table 3, multiple metrics are used to evaluate the position accuracy and convergence time, in which ΔE, ΔN and ΔU are the 95% percentile of position error for each components, the convergence time for each method are determined by the longest convergence time after each re-initialization. Because there are more unmodelled biases in the kinematic mode such as much severer multipath error, the kinematic position accuracy of three methods are generally lower than the static experiments, thus, the horizontal and vertical position error thresholds for determining convergence time are set as 50 cm and 40 cm, respectively. Compared with the SBAS method, the position accuracy can achieve an improvement of at least 20 cm in each positioning component through using the SBAS-PPP method. With the final correction products from IGS, the north and up components of position errors from IGS-PPP can be reduced by 50% and 18%, respectively. The position errors in the converged state can be partially reflected by the root-mean square (RMS) values. It can be found that the position accuracy difference between SBAS-PPP and IGS-PPP becomes smaller than the static experiment. It is partially because the precision of reference positioning value is 5 cm. The corresponding horizontal (CT_H_) and vertical (CT_V_) convergence time for two methods are also listed in the table. Since the position accuracy of SBAS method is too low to achieve the predefined convergence threshold, the convergence time of SBAS-PPP and IGS-PPP are compared. Compared with the proposed SBAS-PPP, we can see that the convergence time can be improved by 27.7 s and 3.6 s for the horizontal and vertical components, respectively, through using the IGS-PPP method. This is because the higher precision of correction products from IGS, and the observation biases have been suppressed more sufficiently. Furthermore, it can be found that the convergence time can be shortened to be less than 500 s, which has shown that the convergence time of PPP can benefit from the change of satellites geometry.

## 5. Concluding Remarks

Real-time single frequency precise point positioning can be performed by using various correction products. Those from SBAS are not intended to be used with PPP, nonetheless, we have demonstrated that the proposed single-frequency PPP method using SBAS correction products can yield the static positioning result at several decimeters level, and the kinematic positioning result at sub-meter level. The position accuracy of SBAS-PPP can achieve a remarkable improvement than SBAS, and a little lower than the single frequency IGS-PPP. However, the proposed SBAS-PPP can be preferable for real-time navigational services due to the real-time availability of correction products. Compared with the traditional SBAS method, the significant position accuracy improvement is observed because more accurate constraints on the ionosphere error are imposed, meanwhile, the SBAS recommended estimation models are also incorporated with the accurately characterized stochastic model. The position accuracy, produced in real-time by using the single-frequency observations in the kinematic experiment, indicates the promising future of using the proposed SBAS-PPP method for high-precision navigation applications with single-frequency receiver.

It is noted that the convergence time of proposed SBAS-PPP method is relatively long, normally several hundreds of epochs to achieve a few decimeters in the static mode, which is worse than the single frequency PPP using IGS products because there are more unmodelled observation biases for the SBAS-PPP and lower precision of SBAS correction products. The issue of over-long convergence time of SBAS-PPP can partially be resolved in the kinematic mode. This is also imply the feasibility of utilizing the proposed SBAS-PPP method for high precision real-time services. In order to get a more general performance of the proposed PPP method, extensive experiments have to be conducted. The performance of the proposed SBAS-PPP is tested with EGNOS. The future work can focus on the investigation of using corrections from other SBASs, such as WAAS, MSAS, and SDCM.

## Figures and Tables

**Table 1:** Processing strategy for real-time single frequency PPP.

Errors	Settings
Satellite orbit and clock error	SBAS real-time satellite products
Ionosphere error	SBAS ionosphere correction as the observation, the parameters [*I_z_* *g_e_* *g_n_*] of the ionosphere model is estimated
Troposphere error	The zenith delay of wet troposphere residual is modeled as the first-order Markov random walk
Phase windup	Phase windup correction proposed by [30]
Solid earth tide	Solid earth tide correction proposed by [3]
Sagnac and relativistic effects	Estimation model recommended by IS-GPS-200
Other estimated parameters	Receiver coordinates, receiver clock error, integer ambiguities

**Table 2:** Statistical positioning results in east-north-up components of 12 stations by using different single frequency positioning algorithms.

Stations	SBAS			IGS-PPP			SBAS-PPP		
	ΔE/cm	ΔN/cm	ΔU/cm	ΔE/cm	ΔN/cm	ΔU/cm	ΔE/cm	ΔN/cm	ΔU/cm
BZRG	48	90	159	4	3	5	3	18	37
HERT	33	86	126	3	3	4	11	26	25
HUEG	63	107	169	1	3	2	15	19	12
IENG	68	104	167	5	1	4	5	11	17
LROC	36	82	102	7	5	5	12	19	18
MATE	52	64	111	9	6	5	4	5	55
OAK2	69	124	170	4	4	4	20	23	47
PENC	43	89	119	5	2	8	5	16	31
PTBB	109	169	285	4	2	6	12	24	24
WARN	68	122	220	5	2	5	5	25	21
YEBE	92	97	200	2	3	1	22	11	39
WTZR	53	101	178	3	4	4	4	19	15

**Table 3:** Kinematic position accuracy statistics using different positioning methods.

	SBAS	IGS-PPP	SBAS-PPP
ΔE/cm	69	38	38
ΔN/cm	87	26	52
ΔU/cm	138	50	61
RMS_E_/cm	53	20	24
RMS_N_/cm	50	12	22
RMS_U_/cm	71	25	32
CT_H_/s	N/A	473.6	501.3
CT_V_/s	N/A	481.2	484.8
